# Resonating Shell: A Spherical-Omnidirectional Ultrasound Transducer for Underwater Sensor Networks

**DOI:** 10.3390/s19040757

**Published:** 2019-02-13

**Authors:** Sina Sadeghpour, Sebastian Meyers, Jean-Pierre Kruth, Jozef Vleugels, Michael Kraft, Robert Puers

**Affiliations:** 1 Department of Electrical Engineering (ESAT-MICAS), KU Leuven, Leuven 3001, Belgium; michael.kraft@kuleuven.be (M.K.); puers@esat.kuleuven.be (R.P.); 2 Department of Mechanical Engineering, KU Leuven, Leuven 3001, Belgium; sebastian.meyers@kuleuven.be (S.M.); jean-pierre.kruth@kuleuven.be (J.-P.K.); 3 Department of Materials Engineering, KU Leuven, Leuven 3001, Belgium; jozef.vleugels@kuleuven.be

**Keywords:** ultrasound transducer, piezoelectric, spherical-omnidirectional, PZT, underwater sensor network (USN)

## Abstract

This paper presents the design and fabrication process of a spherical-omnidirectional ultrasound transducer for underwater sensor network applications. The transducer is based on the vibration of two hemispheres with a thickness of 1 mm and an outer diameter of 10 mm, which are actuated by two piezoelectric ring elements. Since the ultrasound wave is generated by the vibration of the two hemispheres, a matching layer is not required. Silicon Carbide (SiC) is used as the material of the hemispherical shells of the transducer. The shells were fabricated by laser sintering as an additive manufacturing method, in which the hemispheres were built layer by layer from a powder bed. All manufactured transducers with an outer dimension of 10×14.2 mm and a center frequency of 155 kHz were measured in a water tank by a hydrophone or in mutual communication. The circumferential source level was measured to vary less than 5dB. The power consumption and the insertion loss of the transducer, ranging from 100 μW to 2.4 mW and 21.2 dB, respectively, along with all other measurements, prove that the transducer can transmit and receive ultrasound waves omnidirectionally at tens of centimeters intervals with a decent power consumption and low actuation voltage.

## 1. Introduction

Following the introduction of Internet of Things (IoT) as a technological revolution of computing and communications to interconnect with terrestrial applications, Underwater Sensor Networks (USN) or more recently, Internet of Underwater Things (IoUT) [1], were adopted to tackle many scientific, industrial and military underwater activities [2,3]. An USN is defined as a myriad of smart underwater sensor nodes, physically distributed near the objects under investigation, and able to communicate wirelessly in a network [4,5]. USNs are envisioned to enable applications for oceanographic data collection, pollution monitoring, assisted navigation [5], aquarium observation, harbor security, tactical surveillance applications [1], pipeline inspection, etc. To render these applications viable, it is mandatory that the submerged devices are equipped with some form of communication. Because of the low absorption of ultrasound waves compared to electromagnetic waves in dense media [6], ultrasound became the dominant technology in underwater communication.

For specific USN applications, such as the sensory exploration of hard-to-reach underwater or in-oil environments, small size nodes with limited power budget are indispensable [7]. The main objective for these applications is to provide devices that can operate in water distribution systems or other hard-to-reach environments [8]. The first two main applications investigated are oil or water pipeline inspection to find obstructions, leaks or faults, and underground channels exploration, in which current technology cannot provide access without damage. It is clear that to access these environments, extreme system constraints are required, such as a miniaturized size for each node, the ability to run on a limited power budget, and the integration of the circuitry in CMOS technology. Therefore, the ultrasonic communication must be realized by a transducer that is small in size (<2 cm in diameter) and is actuated by an embedded CMOS circuit that is compatible with the supply voltage (<5 V) to allow a communication range of 1–2 m.

Another restriction that makes it difficult for USNs to explore hard-to-reach environments is the unpredictable nature of the environment they explore. For instance, due to the existence of substantial turbulence and chaotic movement of the sensor nodes in pipelines, it is almost impossible to predict the direction and position of the individual nodes while performing their measurement tasks. Therefore, to enable acoustic communication between two sensor nodes, the acoustic signal must be sent in all directions in space to allow the signal to reach the nearby sensor node. In the words, the sensor node used in USN must be able to perform ultrasonic communication in a full spherical-omnidirectional beam pattern while housing the driving electronics and the powering source. Commercial ultrasound transducers which are based on a vibrating disk or surface, such as a PZT disk vibrating in thickness mode, are only partially omnidirectional in the space to which the frontal area of the transducer is targeting.

In the following paragraph, we highlight some cutting-edge spherical-omnidirectional ultrasound transducers in the literature and market.

One of the first architectures of a spherical-omnidirectional ultrasound transducer consists of a spherical surface covered by multiple individual ultrasound transducers. For instance, Yang et al. introduced a large spherical transducers array in which commercial transducers are placed on the surface of a sphere [9]. In a similar manner, Nishitani et al. manufactured a spherical-omnidirectional ultrasound transducer by placing several transducers on the surface of an icosahedron object [10]. In a more complex way, Zhou et al., Chen et al. and similarly Wildes et al. covered the tip of a catheter with diced thick piezoelectric materials for spherical-omnidirectional bio-imaging applications [11,12,13]. Recently, Mimoun et al. [14] introduced a generic platform for the integration of sensing micromechanical devices at the tip of a medical instrument, such as a catheter, to obtain a spherical beam pattern. As an industrial example, the spherical transducers from Benthowave instrument Inc., Sea, Reson, and Sensor technology Ltd. can be referred to. These commercial spherical-omnidirectional transducers are based on spherical PZT shells, which are very costly to be fabricated. Moreover, these spherical PZT shells have a very large parasitic capacitance, which leads to a very high power consumption.

All aforementioned transducers are distinguishable by two categories; transducers based on micro-electromechanical technology, and transducers assembled by discrete components. These two categories, as transducers used in USN, have some issues:

- Transducers based on micro-electromechanical technology: These transducers require a complex fabrication process. Moreover, in the most cases their covering distance range is limited to few millimeters.
- Transducers assembled by discrete components: This category of transducers has either a very big dimension or high cost of assembly.

Furthermore, in both categories most of the transducers require a very high actuation voltage in the range of a few tens of volts [15]. For instance, the spherical transducer from Benthowave instrument Inc. requires 100–300 V${}_{\mathrm{rms}}$ actuation voltage.

In this paper, we introduce a novel ultrasound transducer for USN applications with a true spherical-omnidirectional beam pattern and a small size based on PZT ring actuators. It features a hollow space to lodge the driving, communication, and sensing electronics inside. The small dimension, and consequently, the limited available power budget demands for a transducer that can operate at a low actuation voltage (CMOS compatible) and with a low power consumption. As the name of the transducer, *resonating shell*, suggests, the source of the ultrasound wave is the vibration of two hemispherical shells that form the housing of the transducer. Therefore, our transducer is an ultrasound generator and cannot be considered as a transmitter. The resonance of each shell is generated by a single PZT piezoelectric ring element. The shell is made of silicon carbide (SiC). For the fabrication of the ceramic shells of the transducer with its complex hemispherical geometry, additive manufacturing (AM) was selected. AM provides flexibility with respect to the geometrical design of the *resonating shell*, as changes can be incorporated rapidly and without extra costs. Additionally, AM can produce prototypes for testing at very low lead time [16].

## 2. Concept and Design Parameters

**Figure 1:**
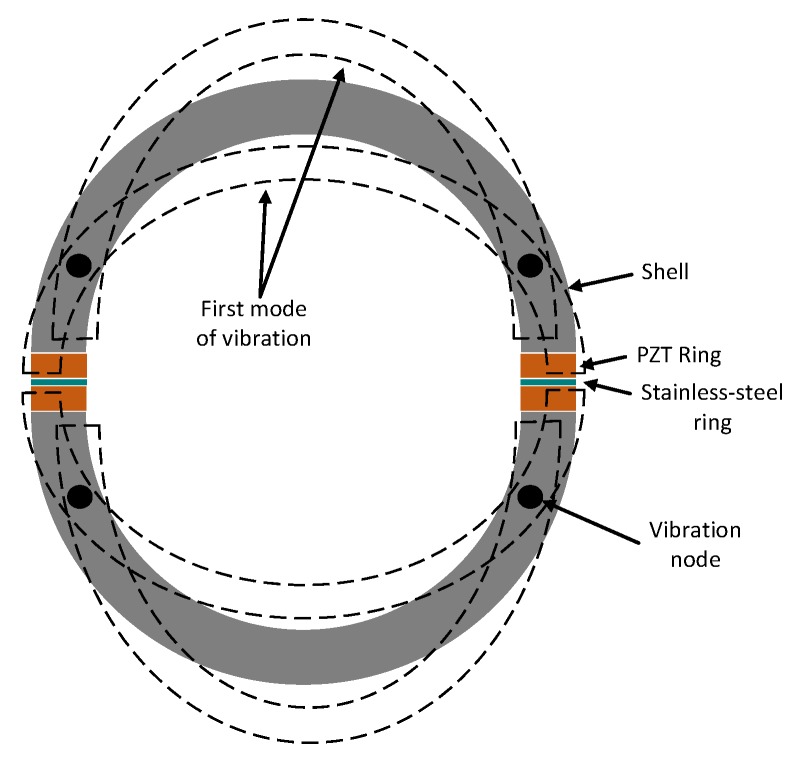
The 2D model of the transducer and its first mode of vibration.

The proposed transducer will be used for underwater sensor network applications with a spherical-omnidirectional beam pattern. Hence, two separate hemispherical shells were chosen as the geometry of the transducer to obtain an omnidirectional radiation when the shells are vibrating at their first resonance mode. To simplify the vibration problem, the transducer is modeled as a 2D structure as shown in Figure 1.

Two half-circles correspond to the hemispherical shells and the extreme positions of each half-circle in the first mode of vibration are shown by dotted lines. The transducer consists of two piezoelectric ring elements and one thin stainless-steel ring. Ring shaped PZT elements were mandatory to enable the required space for the driving electronics and its power source. When the piezoelectric elements on both ends of each half-circle vibrate, the whole structure can be driven into resonance. The resonance frequency is defined by Equation (1) [17]

(1) $$f_{b}=\frac{\gamma^{2}}{2\pi a^{2}}\sqrt{\frac{EI}{\rho A}}$$

where *E* is the Young’s modulus, ρ the density per unit of length, *I* the moment of inertia of the cross section, *a* the radius of the hemisphere, *A* the area of the cross section, and γ a coefficient defined by the mode of vibration. Whereas Equation (1) describes the resonance frequency of the simplified 2D structure, Equation (2) can be interpreted as the relationship between the resonance frequency of a spherical structure and its diameter (*a*), density (ρ), thickness (*t*), and Young’s modulus (*E*). It should be noted that Equation (2) is only valid for a spherical shell. Since the *resonating shell* is not a single material but consists in a combination of PZT and stainless-steel parts, the real resonance frequency is slightly different from what Equation (2) suggests.

(2) $$f_{h}∼\frac{t}{a^{2}}\sqrt{\frac{E}{\rho }}$$

**Figure 2:**
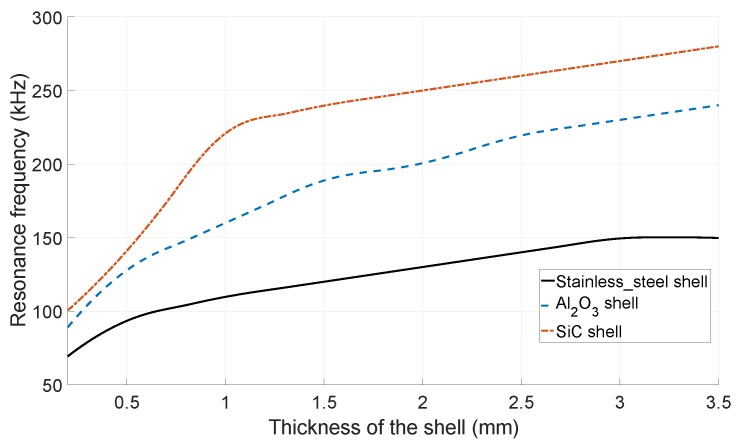
The simulated first mode resonant frequency of the *resonating shell* transducer with three different shell’s materials.

As a comparison between different shell’s materials, Figure 2 shows the FEM simulation results of the first mode resonant frequency of the transducer, with a 10 mm outer diameter, as a function of the shell’s thickness (consisting of stainless-steel, aluminum oxide, and silicon carbide). The simulation was performed by COMSOL Multiphysics 5.3 (Comsol Ltd., Stockholm, Sweden) with default material parameters. As Equation (2) suggests, the resonance frequency of the stainless-steel and aluminum oxide shells is lower than for SiC, due to their lower Young’s modulus to density ratio. Indeed, a low resonance frequency reduces the bit-rate of the used communication protocol. SiC, because of its high Young’s modulus to density ratio, is a good candidate to obtain a sufficiently high resonance frequency. Since the input electrical capacitance of the transducer depends on the interface area of the hemispherical shells and PZT elements, a tick shell results in high input electrical capacitance, and, consequently a high power consumption. Hence, to compromise between resonance frequency and input electrical capacitance, 1 mm was selected as the thickness of the shells, whereas a thicker shell does not result in a significant higher resonance frequency, as can be seen in Figure 2. Furthermore, choosing a shell thickness of 1 mm results in a spherical empty space with a diameter of 8 mm inside the transducer, which is intended to house the interface electronics and its power source.

**Figure 3:**
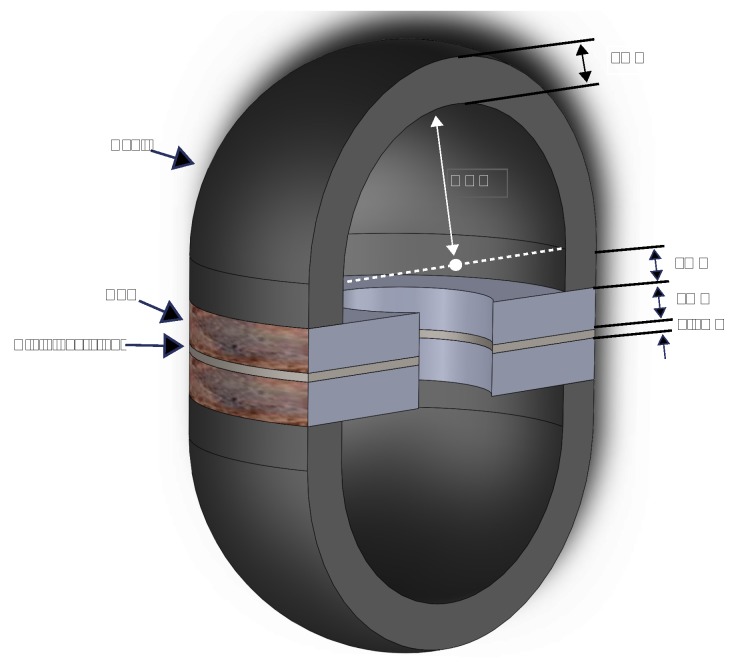
The final architecture and dimensions of the *resonating shell* transducer.

The final architecture of the transducer is shown in Figure 3. The two PZT elements have opposite polarity, enabling the whole spherical body to vibrate, as shown in Figure 1. Similar to the 2D model, each hemisphere in the transducer has one node of vibration in its first resonance mode, which basically is static and generates minimum pressure in the working medium. To improve the omnidirectionality of the transducer, one may extend and elongate the end of each hemispherical shell to shift the vibration node to the extended part. In this case, the vibration of the extended part with respect to the other parts of the transducer shell has a 90 degrees phase difference. To not affect the resonance frequency by making this extended part too long, 1 mm was chosen to be added to the end of each hemisphere.

A thin stainless-steel layer was placed in between each piezoelectric element to damp the acoustic wave. Furthermore, the stainless-steel layer is static with respect to the other parts of the transducer, since the net value of all applied forces on it is equal to zero. This helps to distinguish the functionality of the hemispheres from each other. The final dimensions of each part of the transducer are shown in Table 1.

## 3. Fabrication and Characterization

### 3.1. Additive Manufacturing of SiC Hemispherical Shells

To fabricate the ceramic hemispherical shells proposed in this work, direct laser sintering (LS) was used. LS is an AM method in which a 3-D geometry is built up layer by layer starting from a powder bed. LS has been proven to be able to produce fully dense reaction bonded carbide ceramics with good mechanical properties and in complex shapes starting from very cheap base materials in powder form. A single powder layer is paved on a supporting plate and is then selectively scanned by a laser. Heat fuses the particles by either completely or partially melting the powder and then leaving it to re-solidify. When the first layer is scanned, a second powder layer is paved on top and laser scanned again. These steps are repeated until the final 3-D part is obtained. AM methods, such as LS, have the advantage of being able to produce complex geometries “for free”, meaning no extra costs are encountered when the part complexity increases.

**Figure 4:**
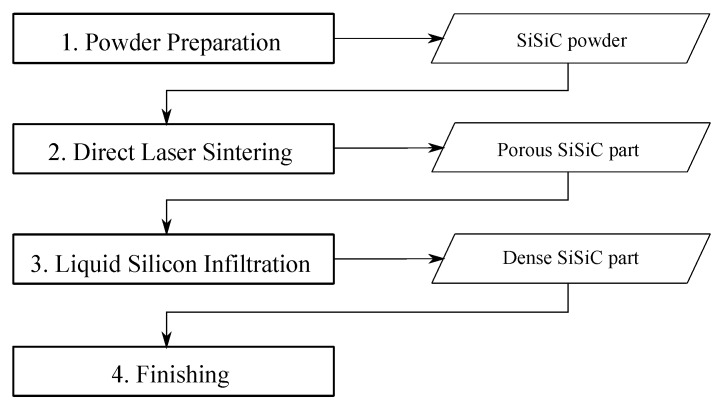
The 4-step approach to produce ceramic hemispheres using additive manufacturing.

Here, this LS technique is used as the shaping method for the transducer hemispheres in ceramic material by the powder metallurgic (PM) process. The PM process is schematically shown in Figure 4. The material used in this work are silicon infiltrated silicon carbide (Si-SiC). The fabrication starts by mixing 40 vol% Si powder (SIMET 985, Keyvest, d${}_{50}$ = 45 μm) with either 60 vol% of α-SiC (CARBOREX BW F320, Washington Mills, d${}_{50}$ = 29 μm) powder in a polyethylene container on a Turbula mixer for 6 h at 75 rpm. This results in powder blends that serve as the starting material for LS. The LS was done under inert Argon atmosphere on a commercial MlabR machine (Concept Laser GmbH, Germany) equipped with a 100 W continuous fiber laser with a spot size ($1/e^{2}$) of 50 μm. During LS, the Si melts and re-solidifies, binding the SiC particles together. The process parameters are summarized in Table 2. Laser sintering results in porous preform hemispheres which are then densified by liquid silicon infiltration (LSI). LSI was done in vacuum (≈0.1 mbar) in a hot press furnace (W100/150-220050 LAX, FCT Systeme, Frankenblick, Germany). The laser-sintered hemispheres were placed in a graphite crucible together with Si powder (Resitec, d${}_{50}$ = 5 μm)). The hot press was heated at 50 °C/min up to 1500 °C with a holding time of 30 min before cooling naturally to room temperature. The Si powder in the crucible melts and infiltrates the pores in the laser-sintered parts, resulting in fully dense Si-SiC hemispheres. These hemispheres were then finalized by a flat grinding of their bottom surface to match the surface of the PZT elements.

### 3.2. Material Characterization of the Shells

Various measurements were performed on the laser-sintered and finished parts to assess the quality and properties of the produced materials. Optical microscopy (VHX-6000 digital microscope, Keyence, USA) was performed to assess the microstructure of both the laser-sintered and infiltrated densified material. Image analysis was obtained using ImageJ software to quantify the amount of residual Si. X-ray diffraction was used (D2 Phaser, Bruker corporation, Billerica, Massachusetts, MA, USA) to investigate the constituent phases in the materials. The density of the materials was measured using the Archimedes method (Acculab atilon ATL-244-1). The Young’s modulus was assessed using the non-destructive impulse excitation method (IET, RFDA, IMCE, Genk, Belgium).

### 3.3. Integration of the Transducer

**Figure 5:**
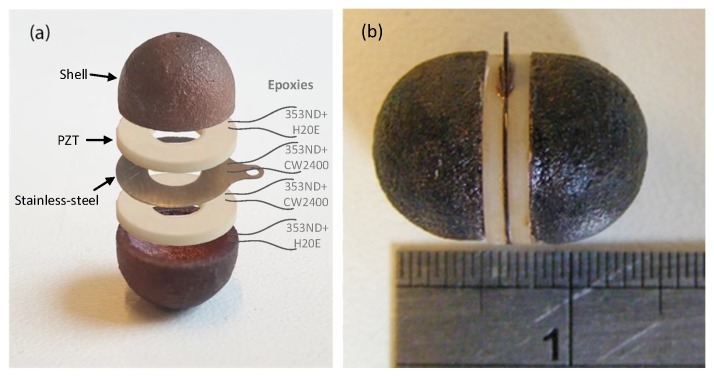
The components of the transducer before integration (**a**) and after integration as a functional transducer (**b**).

As shown in Figure 5a, the *resonating shell* transducer consists out of five components: two hemispherical shells, one stainless-steel, and two PZT rings (Physik Instrumente (PI) GmbH & Co, Karlsruhe, Germany). The parts of the transducer were assembled by means of conductive and non-conductive epoxies. Three epoxies, Epotek H20E, Circuit Works CW2400, and Epotek 353ND were used in the integration of the transducer, among which the first two epoxies are electrically conductive, whereas the last one is a non-conductive and medium viscose epoxy. Conductive epoxies were used in between the PZT/stainless-steel and PZT/shell to use the stainless-steel and the shells as bottom and top electrode, respectively, since the Si-SiC hemispherical shells are conductive. Epotek 353ND was always used in the combination with conductive epoxies to ensure the hardness, smoothness, and the uniformity in the epoxy slurry.

**Figure 6:**
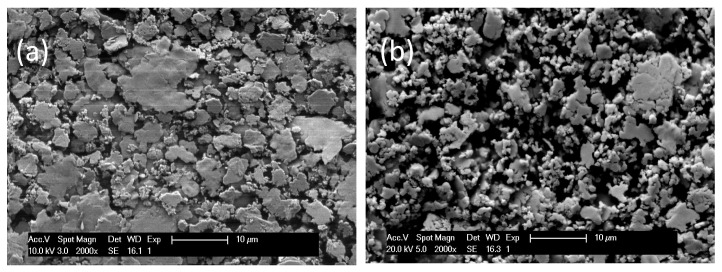
SEM image of the conductive silver epoxy Epotek H20E (**a**) and Circuit Work CW2400 (**b**).

Figure 6 shows the SEM image of the conductive silver epoxies with comparable silver particle sizes. The porosity of CW2400 and H20E is 45% and 18%, respectively.

**Figure 7:**
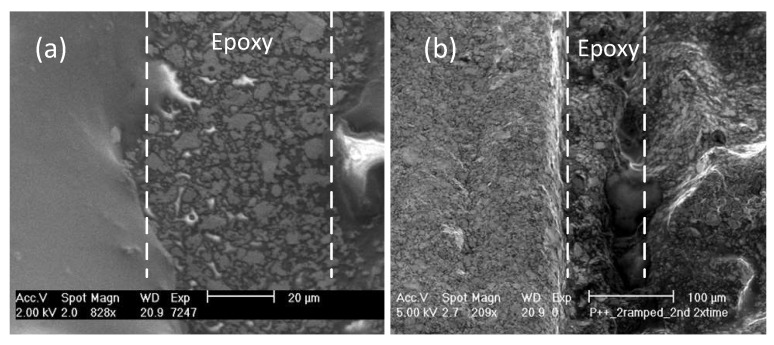
SEM image of the CW2400 epoxy at the interface of the PZT element and the stainless-steel layer with (**a**) and without (**b**) the mixture of 353ND epoxy.

In the first step of the *resonating shell* integration, the PZT elements and stainless-steel layer were assembled. Therefore, CW2400, due to its high porosity, in combination with 353ND, was used as the backing epoxy between each PZT element and the stainless-steel layer to damp the acoustic waves as much as possible. At the same time, the conductivity and hardness at the interface of the PZT elements and the stainless-steel layer could be kept sufficiently high. This guarantees that the stainless-steel layer can be used as the bottom electrode of the transducer. Figure 7 shows a SEM image of the interface between PZT and stainless-steel with and without using 353ND in combination with CW2400. As shown, the use of 353ND ensured the smoothness and uniformity. After applying the epoxies by a micro-dispenser, the sandwiched layers (PZT/stainless-steel/PZT) were slightly pressed and kept in an oven at 110 °C for 10 min.

The second step is the assembly of the hemispheres to the PZT/stainless-steel/PZT component. In order not to alter the acoustic impedance of the hemispherical shell seen by the PZT element, the thickness of the epoxy layer used at the interface of the PZT and the shell was kept as thin as possible. This is explained by Equation (3) [18], where $Z_{in}$ is the acoustic impedance seen by the PZT element, $Z_{s}$ the acoustic impedance of the shell, $Z_{e}$ the characteristic acoustic impedance of the epoxy, *t* the thickness of the epoxy, and *k* the wavenumber and is equal to 2π/λ, and λ the wavelength.

(3) $$Z_{in}=Z_{e}\frac{Z_{s}\cos\left(\beta l\right)+jZ_{e}\sin\left(\beta l\right)}{Z_{e}\cos\left(\beta l\right)+jZ_{s}\sin\left(\beta l\right)}$$

**Figure 8:**
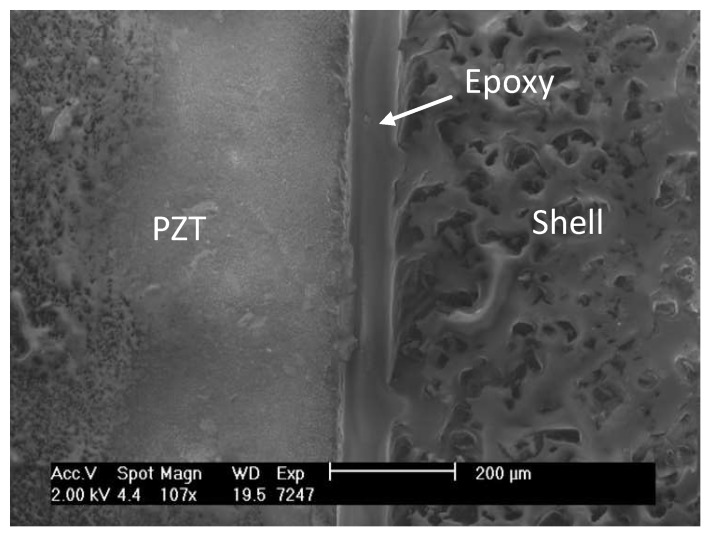
SEM image of the thin epoxy layer between the shell and PZT element.

If *l* converges to zero, then $Z_{in}$ converges to $Z_{s}$. After dispensing H20E in combination with 353ND in between the PZT elements and shells, they were clamped together and pressed to minimize the thickness of the epoxy interface. Thereafter, they were placed in an oven at 175 °C for 10 min to cure. The result is shown in Figure 8. The positions where the epoxies were applied and the fully assembled transducer are shown in Figure 5a,b, respectively.

**Figure 9:**
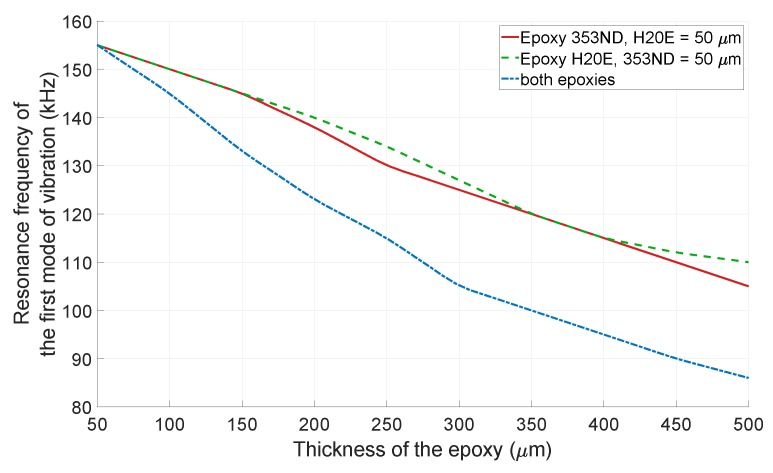
The FEM simulation of the resonance frequency of the first mode of vibration with respect to the thicknesses of the 353ND and H20E epoxies.

The thickness variation of the epoxies may alter the resonance frequency of the *resonating shell*, due to the low value of the Young’s modulus with respect to the PZT element and SiC shell. A thick epoxy layer damps the vibration of the PZT element and reduces the resonance frequency. To investigate the effect of the epoxy thicknesses on the resonance frequency in more details, a FEM simulation was performed. The epoxy between the PZT element and the SiC shell was assumed to be Epotek 353ND with a Young’s modulus of 3.5 GPa, and the Epotek H20E was considered as the epoxy between the PZT element and the stainless-steel layer with a Young’s modulus of 5.57 GPa [19,20]. Figure 9 shows the resonance frequency of the first mode of vibration, while the thicknesses of the 353ND and the H20E epoxies were varied from 50 μm to 500 μm. As shown, when the thickness of each epoxy increased 20 μm individually, while the thickness of the other epoxy was kept constant and equal to 50 μm, the resonance frequency decreased 1.3%. However, increasing the thicknesses of the both epoxies at the same time for 20 μm resulted in a 2.6% reduction of the resonance frequency.

### 3.4. Characterization of the Transducer

**Figure 10:**
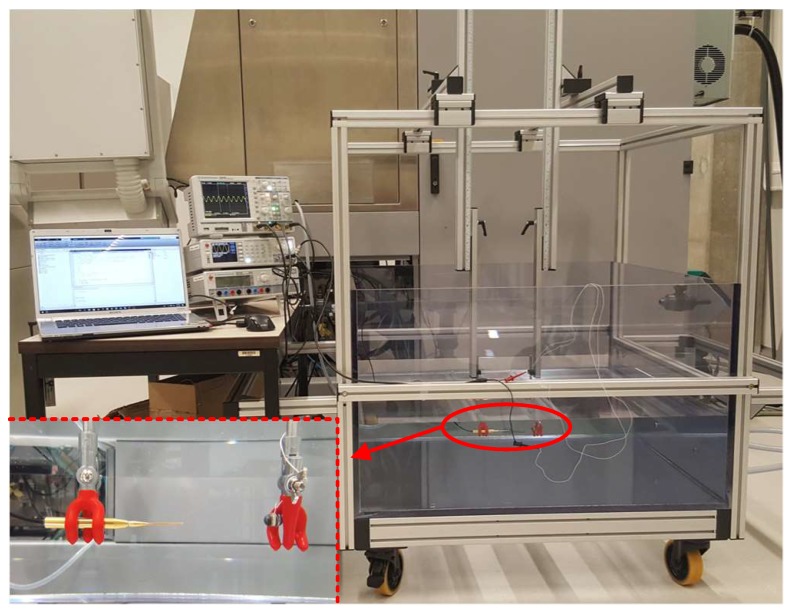
The in-house built measurement setup to characterize the *resonating shell* transducer.

All fabricated *resonating shell* transducers were measured in an in-house built water tank (KU Leuven, MICAS, NanoCentre) with two movable arms in all directions, filled with more than 300 liters deionized water, as shown in Figure 10. Since the applications of the transducer is underwater communication and localization, the measurements were done either by means of a 1 mm needle hydrophone and its preamplifier (Precision Acoustics Ltd., Dorchester, UK or by a mutual communication among two individual transducers. The transducers were actuated by burst sinus waves from an arbitrary wave generator (HMF2550, Rohde & Schwarz, Munich, Germany). The measured signal either from the hydrophone or from a second *resonating shell* was recorded by an oscilloscope (HMO2024, Rohde & Schwarz, USA) on 1-MΩ coupling mode and stored and analyzed by a computer. The communication performance among two transducers was characterized by measuring the insertion loss (IL) in the frequency domain. It shows the resonance mode at which the communication can be performed. If the input and output resistance over two measured transducers are considered to be equal, IL can be represented by Equation (4) [11], in which $V_{a}$ and $V_{r}$ are actuation voltage of the transmitter transducer and received voltage of the receiver transducer, respectively. In order not to affect the measured IL by the loss of channel, the transducers were place in their Rayleigh distance from each other [21].

(4) $$IL=20\log\left(\frac{V_{a}}{V_{r}}\right)$$

## 4. Results and Discussion

### 4.1. Additively Manufactured Shells of the Transducer

**Figure 11:**
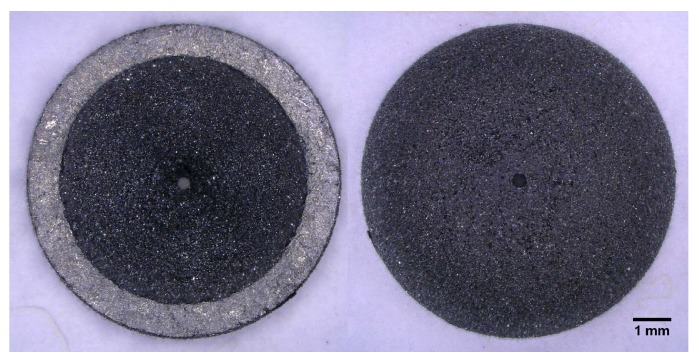
Optical microscopy of the laser-sintered hemisphere, bottom (**Left**) and top (**Right**).

**Figure 12:**
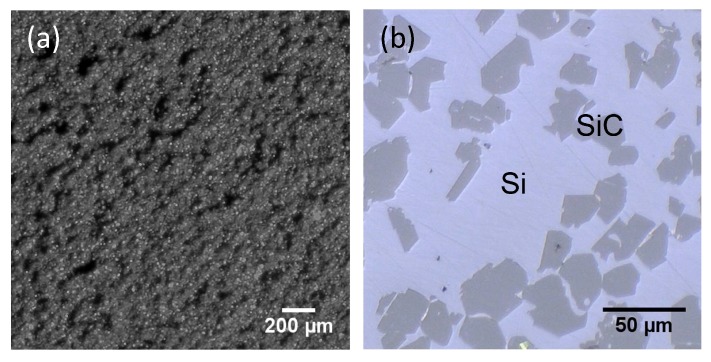
Optical microscopy of the laser-sintered (**a**) and infiltrated (**b**) Si-SiC material.

After LS, the Si-SiC parts were subjected to optical microscopy. A laser-sintered hemisphere can be seen in Figure 11. The materials were investigated at higher magnification to gain an idea about the surface morphology of Si-SiC material. The top surface structure is shown in Figure 12a.

**Figure 13:**
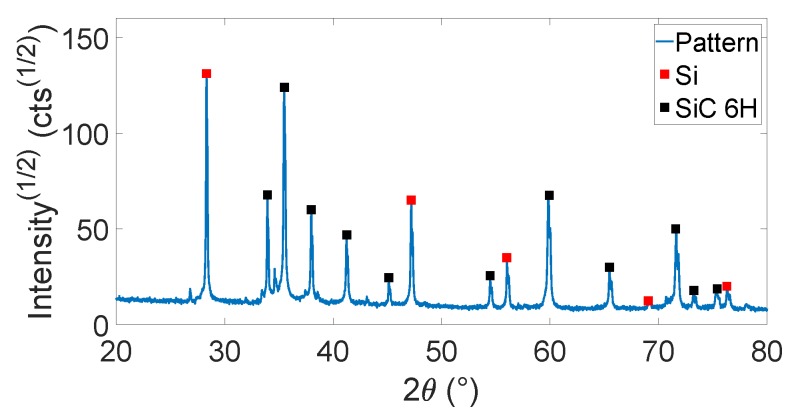
XRD spectrum of the densified Si-SiC material, with peaks corresponding to Si (red) and SiC (black).

The Si-SiC material was then infiltrated with liquid silicon at 1500 °C to fill the porosity and obtain fully dense ceramic materials. This material was subjected to X-ray diffraction (XRD) to verify which phases were present. XRD patterns of the Si-SiC ceramic is shown in Figure 13. The Si-SiC diffraction pattern shows peaks of Si and SiC, as expected.

**Figure 14:**
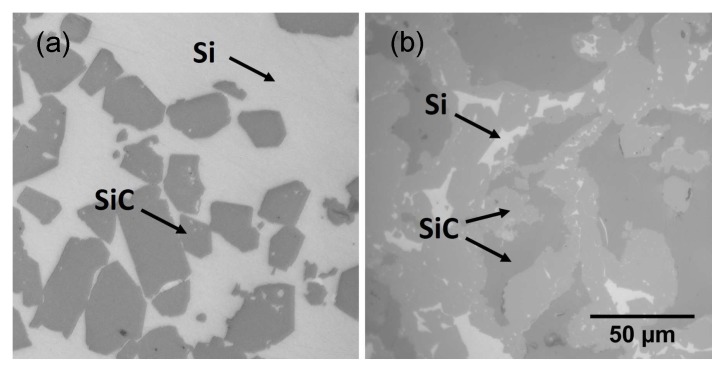
Optical microscopy of the infiltrated Si-SiC (**a**) and phenolic resin impregnated Si-SiC (**b**) materials.

The material was further studied using optical microscopy, as presented in Figure 12b. The Si-SiC composite shows relatively large areas of Si (white) with some dispersed SiC (grey) grains. These SiC grains originate from the starting powder. Image analysis showed a SiC content of 34.7 ± 0.7 vol%, meaning 65 vol% of the material is residual Si. It is important to note that the residual Si content has a negative influence on the mechanical properties of the final material. The Young’s modulus for example, an important characteristic for the *resonating shells*, will decrease with higher Si content. Therefore, the Si content of the Si-SiC material was tuned to a lower level by adding an extra step in the fabrication process. After LS, the Si-SiC material was impregnated with a phenolic resin [22]. This Si-SiC with phenolic resin is then placed in an oven in inert atmosphere to pyrolyze the resin, yielding about 60 wt.% porous carbon with respect to the original weight of the resin. The Si-SiC-C material is subsequently infiltrated with liquid Si. During LSI, the liquid Si reacts with the carbon to form secondary SiC. In this way, the final SiC content can be increased up to 84.9 ± 1.3 vol%, meaning only about 15 vol% of residual Si remains. The microstructure of the obtained material is compared to the standard Si-SiC without additional phenolic resin impregnation in Figure 14. In the phenolic resin impregnated Si-SiC, two different SiC phases can be seen. The dark grey is the SiC originating from the starting powder, whereas the lighter grey phase is the reaction formed SiC.

The two produced materials were further characterized by measuring the density and Young’s modulus, to assess the specific modulus, which is relevant for the final application. The results of these measurements are shown in Table 3. All two materials are suitable for application in the transducer shell.

### 4.2. Resonating Shell as an Ultrasound Transducer

**Figure 15:**
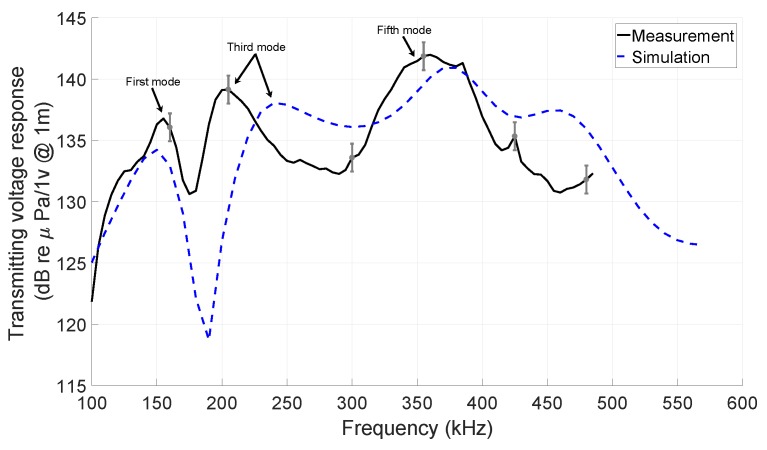
The measured and simulated transmitting voltage response (TVR) with respect to the frequency (Frequency response) of the transducer, which shows the first, second, and higher mode of vibrations.

The functionality of the integrated *resonating shells* was measured under water in communication with another *resonating shell* transducer or a hydrophone. Figure 15 shows the FEM simulation and the corresponding measurement of the transmitting voltage response (TVR) with respect to the frequency of the transducer (Frequency response), which was measured by the hydrophone to visualize all resonance modes in the frequency spectrum. Due to the 14% uncertainty of the hydrophone sensitivity, which has been inscribed in the calibration certificate of the hydrophone, an error bar was added in the Figure 15. The positions of the resonance frequency peaks are in good agreement with the simulation except for the third mode of vibration that mainly results from the differences between the real mechanical parameters of the PZT elements and stainless-steel layer and the ones used in the simulation. The resonance frequency of the first and third mode of vibration were 155 kHz and 205 kHz with TVR value of 137 and 139 dB re μPa/1V @ 1m, respectively. The obtained Q-factor at the first mode of vibration (155 kHz) was 6.2, since the 3 dB bandwidth was 25 kHz. The resonance frequency of the third mode of vibration had four nodes and three lobes on each hemispherical shell, between which there was a 90° phase shift. This phase shift is measurable by turning the hydrophone around the transducer.

**Figure 16:**
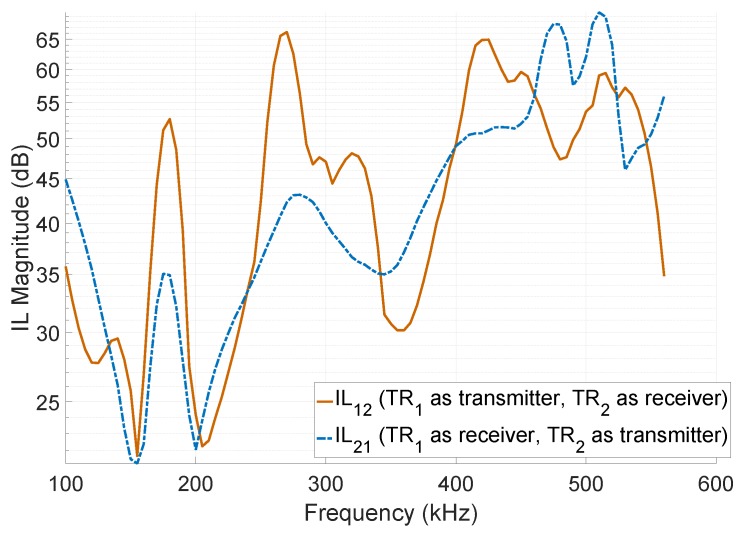
The measured insertion loss (IL) respect to the frequency in the communication between two transducers. The IL was measured one time with the preferential communication direction from the first transducer (TR${}_{1}$) to the second transducer (TR${}_{2}$) and one time from the TR${}_{2}$ to the TR${}_{1}$.

Figure 16 shows the measured IL with respect to the frequency among two transducers. As shown, the minimum IL was obtained at 155 kHz (21 dB) and 205 kHz (22 dB), which are the first and third mode of vibration, respectively. However, the real IL tends to be lower than the measured one, since during the measurement only a fraction of the acoustic power was received by the receiver. This is due to the spherical beam pattern of the *resonating shell*.

As shown in Figure 16, at higher mode of vibrations, the transducer had a lower receive sensitivity with respect to the first and third mode. The third mode of vibration had a worse omnidirectional response than the first mode, due to:

1. There are phase variations around the transducer in the third mode of vibration, which makes it non-ideal for a spherical-communication.
2. At the third mode of vibration, the receiver transducer is only sensitive to the perpendicularly impacted wave on the top part of the shell. The wave that impacts the transducer within a $45^{\circ }$ angle with respect to the axial direction cannot excite the third mode of vibration.

Therefore, 155 kHz, the first mode of vibration, was chosen to be the working frequency.

**Figure 17:**
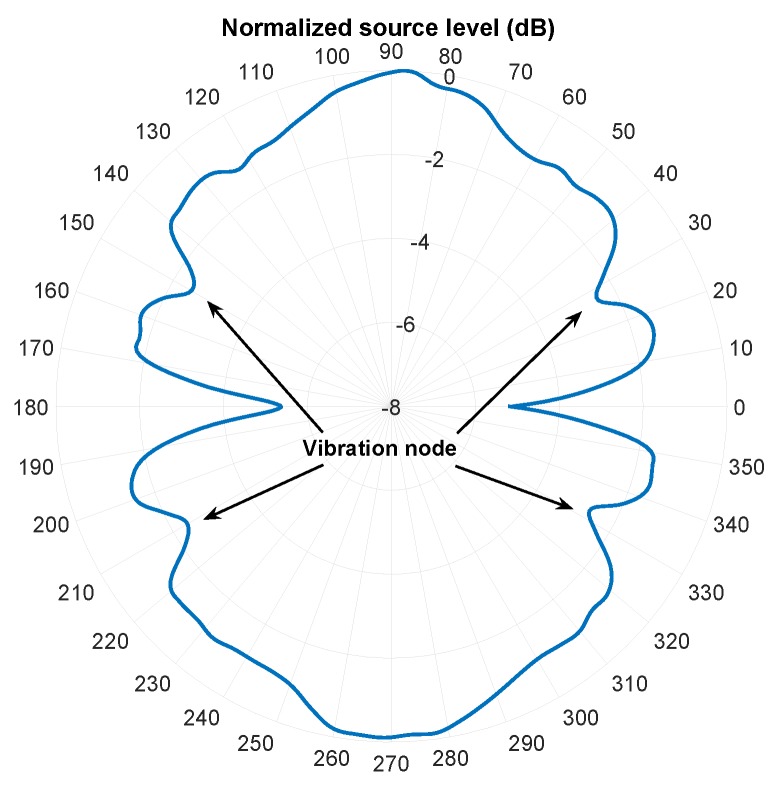
The measured circumferential pressure beam pattern of the transducer at its first mode of vibration (155 kHz). At zero degrees the measurement line is aligned and parallel with the surface of the stainless-steel layer and at 90 degrees it is aligned and perpendicular respect to the top of the transducer’s hemisphere.

The beam pattern was obtained by rotating a transducer around another transducer to measure the source level and normalizing it to the axial source level, which was measured at the front side and on the perpendicular axis to the transducer. The normalized source level is obtained by Equation (5)

(5) $$SL_{n}=20\log\left(\frac{V_{r}}{V_{90^{\circ }}}\right)$$

in which, $SL_{n}$ is the normalized source level, $V_{r}$ the received voltage by the receiver transducer, and $V_{90^{\circ }}$ the axial received voltage. The beam pattern is shown in Figure 17, in which $90^{\circ }$ corresponds to the axial direction of the *resonating shell*. The circumferential source level of the transducer varies less than 5dB, which allows reliable spherical-omnidirectional communication between different transducers at an arbitrary orientation with respect to each other. The 5dB loss in the source level happens at $0^{\circ }$, and at other positions around the *resonating shell* there is not more than 3dB loss. The positions of the vibration nodes are also visible in the beam pattern, which generate less pressure amplitude with respect to the other points around the transducer.

**Figure 18:**
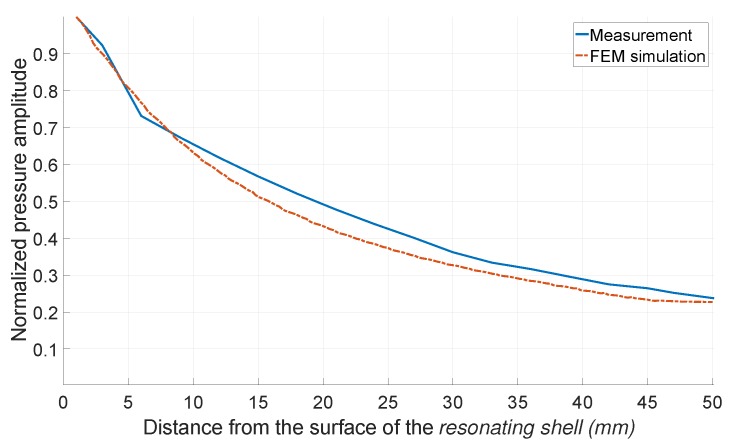
The FEM simulation and measured normalized pressure amplitude of the *resonating shell* in the near field along the axial distance.

It is well known that the pressure intensity of an ultrasound transducer along the beam axis has sinusoidal variations in the near field. The near field can be defined as the Rayleigh distance [21]. To characterize the behavior of the *resonating shell* in the near field, the pressure amplitude was measured at 1 mm intervals from the surface of the transducer and was compared with the simulation result. As shown in Figure 18, the measurement and simulation results confirm that there is less than 0.7% variation in the pressure amplitude in the distance of 10 mm from the surface of the transducer. This confirms that the *resonating shell* is truly a spherical ultrasound source, since the far-field begins from the surface of the transducer. As a result, the whole area around the *resonating shell* can be considered as the usable distance range.

**Figure 19:**
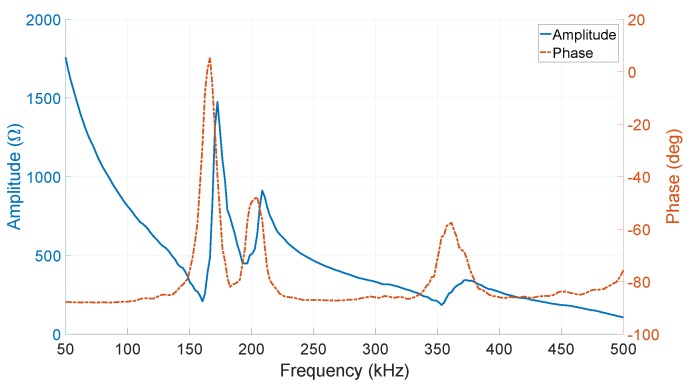
The measured amplitude and phase of the electrical input impedance of the transducer respect to the frequency.

The amplitude and phase of the input electrical impedance of the transducer is shown in Figure 19. The resonance frequency, $f_{r}$ and the anti-resonance frequency, $f_{a}$, are 155 kHz and 175 kHz, respectively. The effective electromechanical coupling factor, which can be calculated by Equation (6) [23,24], was found to be 50%.

(6) $$k_{t}=\sqrt{\frac{\pi }{2}\frac{f_{r}}{f_{a}}\tan\left(\frac{\pi }{2}\frac{f_{a}-f_{r}}{f_{a}}\right)}$$

By knowing the amplitude and phase of the input impedance at the center frequency, the dynamic power consumption of the transducer for a continuous sinus signal, can be calculated by Equation (7)

(7) $$P=\frac{1}{2}\frac{V_{m}^{2}}{Z_{a}}\cos\left(\theta \right)$$

where $V_{m}$ is the peak actuation voltage, and $Z_{a}$ and θ are the input impedance amplitude and phase, respectively. For example, the power consumption of the *resonating shell* for transmitting a continuous sine wave at 1 V${}_{p-p}$, 3 V${}_{p-p}$, and 7 V${}_{p-p}$ is about 97.7 μW, 878.5 μW, and 4.87 mW, respectively.

**Figure 20:**
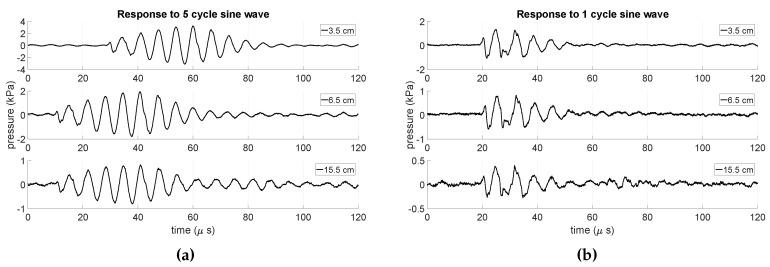
The measured time response of the *resonating shell* transducer to a 7 V${}_{p-p}$ burst sinus signal with 5 cycles (**a**) and 1 cycle (**b**) at different distances.

To characterize the time response of the *resonating shell*, a burst sinus signal was transmitted and measured by a hydrophone. Figure 20 shows the time response of the *resonating shell* transducer to a burst sinus signal with 1 and 5 cycles actuated with 7 V${}_{p-p}$ at different distances, ranging from 3.5 cm up to 15.5 cm. The generated pressure amplitudes by 5 cycles and 7 V${}_{p-p}$ actuation were 3.2, 1.9, and 0.8 kPa at distances 3.5, 6.5, and 15.5 cm, respectively. The obtained transient time, while the *resonating shell* was actuated with a 1 cycle burst sinus signal, was equal to 35 μs. Consequently, in a mutual communication between two *resonating shell* transducers, a transient time of about 70 μs should be taken into account. This means that a maximum communication bit-rate of 14.3 kbit/sec by a simple On-Off Keying (OOK) modulation scheme can be obtained.

Table 4 shows the comparison of the *resonating shell* with respect to some state-of-art and commercial spherical-omnidirectional transducers. Simple design, small dimension, low electrical capacitance, and low actuation voltage are the key factors that distinguish the *resonating shell* from other transducers.

## 5. Conclusions

A spherical-omnidirectional ultrasound transducer, *resonating shell*, for USN applications has been fabricated and characterized. The proposed transducer consists of two piezoelectric PZT ring elements, one stainless-steel supportive layer, and two hemispherical shells, which are made by LS as an AM method. The actuation of the piezoelectric elements leads to the vibration of the hemispherical shells in the first resonance mode (155 kHz) to generate an ultrasound wave with a spherical-omnidirectional beam pattern. In this vibration mode, the circumferential source level varies less than 5dB and the Q-factor, the IL, and the TVR are 6.2, 21 dB, and 137 dB re μPa/1V @ 1m, respectively. The power consumption of the *resonating shell* for transmitting a continuous sinus signal at 3 V${}_{p-p}$ is 878.5 μW. The *resonating shell*, because of its simple fabrication process, small dimension, low actuation voltage, low power consumption, and sufficient TVR and bandwidth, is a promising ultrasound transducer for small underwater sensor nodes with limited power budget. 

## Figures and Tables

**Table 1:** The final dimensions of each part of the transducer.

Property	Value (mm)
Inner radius of the shell	4
Outer radius of the shell	5
The thickness of the stainless-steel ring	0.2
The thickness of the PZT rings	1

**Table 2:** Laser sinter parameters for Si-SiC powders.

Property	Si-SiC
Laser power (W)	15
Scanning speed (mm/s)	100
Layer thickness (μm)	30

**Table 3:** Properties of the three laser-sintered and infiltrated materials.

Property	Si-SiC	Impregnated Si-SiC
Density ρ (g/cm${}^{3}$)	2.57	2.82
Young’s modulus E (GPa)	232	285
E/ρ (MPa/(kg/m${}^{3}$))	90.3	101.1
$\sqrt{E/\rho }$ ($\sqrt{{\mathrm{MPa}/(\mathrm{kg}/m}^{3}}$))	9.5	10.1

**Table 4:** The comparison between the resonating shell and state-of-art and commercial spherical-omnidirectional transducers.

Transducer	Dimension	Resonance Frequency	TVR	Capacitance	Q-Factor	Beam Width	Architecture of the Transducers
*resonating shell*	14.2 × 10 mm	155 kHz	137 dB	4 pF	6.2	360°	2 ring PZTs
Sensor tech (SX series)	50–110 mm Dia	18.5–66 kHz	144 dB	8.4–30 nF	2.6–5.5	—	2 PZT hemispheres
Benthowave (BII-7520 series)	35–80 mm Dia	20–85 kHz	150 dB	—	4	260°–280°	2 PZT hemispheres
Reson TC4033	25 × 80 mm	1–100 kHz	144 dB	7.8 nF	—	270°	2 PZT hemispheres
[9]	195 mm Dia	16 kHz	—	—	—	360°	36 transducers
[10]	> 200 mm	< 40 kHz	—	—	—	360°	60 transducers
